# NLRP3-dependent lipid droplet formation contributes to posthemorrhagic hydrocephalus by increasing the permeability of the blood–cerebrospinal fluid barrier in the choroid plexus

**DOI:** 10.1038/s12276-023-00955-9

**Published:** 2023-03-03

**Authors:** Zhaoqi Zhang, Peiwen Guo, Liang Liang, Shiju Jila, Xufang Ru, Qiang Zhang, Jingyu Chen, Zhi Chen, Hua Feng, Yujie Chen

**Affiliations:** 1 Department of Neurosurgery and State Key Laboratory of Trauma, Burn and Combined Injury, Southwest Hospital, Third Military Medical University (Army Medical University), 400038 Chongqing, China; 2 Chongqing Key Laboratory of Precision Neuromedicine and Neuroregenaration, Southwest Hospital, Third Military Medical University (Army Medical University), 400038 Chongqing, China; 3 Chongqing Clinical Research Center for Neurosurgery, Southwest Hospital, Third Military Medical University (Army Medical University), 400038 Chongqing, China; 4 CAS Key Laboratory of Separation Science for Analytical Chemistry, Dalian Institute of Chemical Physics, Chinese Academy of Sciences, 116023 Dalian, China

**Keywords:** Hydrocephalus, Blood-brain barrier

## Abstract

Hydrocephalus is a severe complication that can result from intracerebral hemorrhage, especially if this hemorrhage extends into the ventricles. Our previous study indicated that the NLRP3 inflammasome mediates cerebrospinal fluid hypersecretion in the choroid plexus epithelium. However, the pathogenesis of posthemorrhagic hydrocephalus remains unclear, and therapeutic strategies for prevention and treatment are lacking. In this study, an Nlrp3^−/−^ rat model of intracerebral hemorrhage with ventricular extension and primary choroid plexus epithelial cell culture were used to investigate the potential effects of NLRP3-dependent lipid droplet formation and its role in the pathogenesis of posthemorrhagic hydrocephalus. The data indicated that NLRP3-mediated dysfunction of the blood–cerebrospinal fluid barrier (B-CSFB) accelerated neurological deficits and hydrocephalus, at least in part, through the formation of lipid droplets in the choroid plexus; these lipid droplets interacted with mitochondria and increased the release of mitochondrial reactive oxygen species that destroyed tight junctions in the choroid plexus after intracerebral hemorrhage with ventricular extension. This study broadens the current understanding of the relationship among NLRP3, lipid droplets and the B-CSFB and provides a new therapeutic target for the treatment of posthemorrhagic hydrocephalus. Strategies to protect the B-CSFB may be effective therapeutic approaches for posthemorrhagic hydrocephalus.

## Brain hemorrhage: Causes of fluid build-up on the brain

Inhibiting a key inflammatory protein and thereby protecting the blood-brain barrier following brain hemorrhage may prevent the build-up of fluid on the brain (hydrocephalus). Using rat models and cell cultures, Yujie Chen and Hua Feng at the Third Military Medical University in Chongqing, China, and co-workers examined how posthemorrhagic hydrocephalus occurs. They found that the acute inflammatory response activated immediately after a brain hemorrhage, which is driven by a critical protein complex involved in innate immunity called the NLRP3 inflammasome, triggers the formation of lipid droplets in the choroid plexus, a part of the brain adjacent to the blood-brain barrier. The lipid droplets interact with mitochondria, increasing the release of reactive oxygen species and damaging the blood-brain barrier. This barrier dysfunction triggers hydrocephalus. Blocking NLRP3 activity improved the function of the barrier following hemorrhage.

## Introduction

Posthemorrhagic hydrocephalus (PHH) is a severe complication and major independent predictor of poor prognosis of intracerebral hemorrhage (ICH), especially ICH with intraventricular hemorrhage (IVH)^1^. Proposed mechanisms for the deleterious effects of IVH include the neuroinflammatory response, possibly caused by blood and its breakdown products^2^. The aim of treatment in ICH patients with IVH (ICH-IVH) and hydrocephalus is to evacuate the intraventricular hematoma, thus relieving the obstruction to cerebrospinal fluid (CSF) flow, reversing ventricular dilatation, and restoring normal intracerebral pressure^3^. However, these invasive operations are highly prone to failure and/or resulting infection^3^. A greater understanding of PHH may improve the treatment and quality of life of ICH-IVH patients worldwide and therefore is essential for developing novel treatment strategies.

The blood-CSF barrier (B-CSFB) plays a crucial role in regulating the spread of many kinds of reactions (such as inflammation and innate immunity) from the periphery to the central nervous system (CNS). The B-CSFB, consisting of choroid plexus epithelial cells (CPECs), acts as a gatekeeper at the brain–immune system interface and maintains homeostasis in the CNS; it also contributes to the pathogenesis and progression of various neurological disorders^4,5^. Adjacent CPECs are bound by tight junctions that form the B-CSFB^6^, but few studies have focused on their function in PHH. CSF hypersecretion was recently demonstrated to be influenced by the activation of ion transporters (NKCC1 and Na^+^/K^+^-ATPase)^7–9^, and it plays an important role in the pathogenesis of hydrocephalus after ICH-IVH.

Lipid droplets (LDs) are microscale organelles located in the cytoplasm and act as hubs for lipid metabolism and energy homeostasis, forming membrane contact sites with multiple organelles^10,11^. Among the proteins closely related to LDs, the most abundant and characteristic is the perilipin (PLIN) family^10^. PLIN3 has been shown to act as a regulatory protein for LD formation in the CNS^12,13^. A recent study indicated that the blood metabolite heme could induce NLRP3-dependent neuroinflammation, lipid peroxidation and neurological deficits^14^. However, the function of CPECs, the primary cells responding to inflammatory injury after ICH-IVH^8,15^, in relation to lipid metabolism has not been explored to date.

We speculated that the acute inflammatory response, especially the NLRP3 inflammasome, contributed to PHH by adjusting the function of the B-CSFB. An Nlrp3^−/−^ rat model of ICH-IVH and primary CPEC culture were used to investigate the potential effects of NLRP3-dependent LD formation and LD function on the pathogenesis of PHH.

## Materials and methods

### Animals

A total of 408 rats were used in the experiments, including 204 adult male wild-type (WT) Sprague–Dawley rats (weight 220–250 g, 8 weeks old), 129 immature WT Sprague–Dawley rats (Experimental Animal Center of Third Military Medical University, Chongqing, China), and 75 adult male Nlrp3^−/−^ Sprague–Dawley rats (Caygen Biological Company, Suzhou, China).

The experimental protocols were approved by the Laboratory Animal Welfare and Ethics Committee of Third Military Medical University (AMUWEC2020762) and performed according to the Guide for the Care and Use of Laboratory Animals. All experiments are reported in compliance with the Animal Research: Reporting of In Vivo Experiments (ARRIVE) guidelines.

In all experimental groups, animals from each litter were randomly assigned to an experimental group prior to the intervention according to a random number table. The experimental operators and observers were blinded to the identity and group of all animals. The data analysts were aware of the animal identity but blinded to the group assignments. Animals were bred in the SPF vivarium and housed under a 12:12 h light-dark cycle with food and water available ad libitum.

### ICH-IVH model and groups

An ICH-IVH rat model was generated by stereotactically guided injection of autologous arterial blood as previously described^16^. Briefly, rats were anesthetized with an isoflurane-oxygen mixture during the surgical procedure and were positioned prone on the stereotactic head. Approximately 200 μl of arterial blood was collected from the femoral artery. A hole (1 mm) was drilled at the designated position (coordinates: 0.2 mm posterior and 2.2 mm lateral to bregma), and a 29-gauge needle on a 1 ml BD syringe was inserted to a depth of 5.0 mm. Next, a total of 200 μl was infused at a rate of 14 μl/min. The sham group was subjected to the same procedure as the ICH-IVH group but without blood injection (only needle insertion was performed). The ICH-IVH (WT) rats were randomly divided into 3 groups: the ICH-IVH, the ICH-IVH+CAY10650, and the ICH-IVH+MitoQ groups. In the ICH-IVH+CAY10650 group, CAY10650 (MCE, USA), an inhibitor of the LD formation–related protein PLIN3, was dissolved in saline and administered at a 10 mg/kg dose by intraperitoneal injection each day after ICH-IVH. In the ICH-IVH+MitoQ group, MitoQ (MCE, USA) dissolved in saline was intraperitoneally administered each day after ICH-IVH (10 mg/kg). The ICH-IVH group received an equal volume of saline as the treatment groups. The details of the experimental design and grouping are described in the Supplementary Material.

### Open-field test

Motor function and exploratory activity were examined in the open-field test. The testing apparatus was a 100 × 100 cm square arena. A video camera suspended above the arena recorded spontaneous motor activity over 5-min trials. Rats were placed in the center of the arena; the total distance traveled and the average motor speed were recorded as indices of motor function, while the amount of time spent in the center was recorded as an index of exploratory activity.

### Modified neurological severity score

The modified neurological severity score (mNSS) was used to comprehensively evaluate neurological function, including motor, sensory and balance functions. Neurological function was graded on a scale of 0–18 (severe injury: 13–18; moderate injury: 7–12; and mild injury: 1–6).

### Western blot analysis

The prepared tissues and cultured cells were solubilized in sample buffer, and the protein concentration of each sample was determined using a Beyotime protein assay kit with bovine serum albumin as the standard. Western blot analysis (30 μg of protein per lane) was conducted using 4–12% SDS gradient polyacrylamide gel electrophoresis followed by a standard blotting procedure. The primary antibodies used included an anti-NLRP3 antibody, anti-PLIN3 antibody, anti-ZO-1 antibody, anti-claudin-5 antibody, and anti-MMP9 antibody. The results were analyzed with ImageJ (National Institutes of Health, Bethesda, Maryland, USA).

### Transmission electron microscopy

Rats were anesthetized by intraperitoneal injection of pentobarbital and then sequentially perfused with normal saline and 4% PFA solution. The choroid plexus was dissected out and kept in 25% glutaraldehyde at 4 °C for 24 h. The prepared choroid plexus was washed in 0.1 M PBS 3 times for 10 min and then treated with 1% OsO_4_ for 2 h. The samples were stained with uranyl acetate and lead citrate after dehydration using an acetone gradient. Finally, the samples were imaged by transmission electron microscopy (TEM; JEM-1230, Japan) and analyzed with ImageJ.

### Transcriptome and proteome sequencing

Choroid plexus samples obtained from different treatment groups, including the sham group, ICH-IVH(WT) group, and ICH-IVH(Nlrp3^−/−^) group, were used for sequencing. Transcriptome sequencing and proteome sequencing were performed by LC-Bio, China.

### Transcriptome sequencing

Briefly, for RNA-seq, total RNA was isolated and purified using TRIzol reagent. The RNA integrity was assessed by an Agilent 2100 with a criterion of an RNA integrity number (RIN) > 7.0. Next, poly(A) RNA I was purified from total RNA and then reverse-transcribed to generate cDNA. After PCR amplification, the cDNA library was constructed. Finally, we performed paired-end sequencing on an Illumina HiSeq-4000 (LC-Bio, China) following the manufacturer’s protocol.

### Proteome sequencing

For proteome sequencing, prepared tissues from different groups were homogenized to powder in liquid nitrogen, and the subsequent proteome analysis was performed by LC-Bio using tandem mass tag (TMT) technology. Briefly, each prepared peptide sample was labeled using TMT reagent. Next, the peptide was fractionated by reversed-phase chromatography. Finally, the sequencing results were obtained through mass spectrometry analysis. Proteins with a fold change>1.2 and *p* value (Student’s *t* test) < 0.05 were considered differentially expressed proteins.

### Bioinformatic analysis

For differentially expressed genes and proteins, gene set enrichment analysis (GSEA) was performed with the standard GSEA tool. Gene sets of interest were generated after different treatments. Gene sets were considered significantly enriched if they had a false discovery rate (FDR) *q*-value <0.25, a |normalized enrichment score| value >1 and a *p* value < 0.05. The network images were analyzed with STRING (functional protein association networks) and Cytoscape.

### Mitochondrial ROS measurement and JC-1 staining

Mitochondrial ROS were measured with MitoSOX Red, a fluorogenic indicator of superoxide explicitly generated by mitochondria. For in vivo mitochondrial ROS analysis, the choroid plexus freshly separated from the brain was cultured in DMEM with 10% FBS for 1 h. Then, 5 μm MitoSOX Red was added to the medium. The mitochondrial membrane potential was measured by JC-1 staining, and the procedure was conducted according to the manufacturer’s instructions (MCE, China). After treatments, the primary CPECs were washed three times with PBS and then incubated with JC-1 staining solution for 20 min at 37 °C. Finally, the stained cells were visualized under a fluorescence microscope for observation and analysis (Zeiss, Germany).

### Cell culture and drug administration

Primary CPECs were cultured as described in previous studies^17^. At P14, rat brains were removed, homogenized and resuspended in 10 ml of sterile dissection medium to obtain the choroid plexus. The choroid plexus was dissociated in CPEC medium and plated on poly-d-lysine- and laminin-coated plasticware for culture. The cells were treated with 1 μg/ml LPS for 12 h. For the intervention groups, MCC950 (5 μm) or CAY10650 (5 μm) was added 1 h after LPS stimulation.

### MRI and ventricular volume analysis

Rats were imaged using a small-animal MRI coil (7 T) under isoflurane anesthesia. T2-weighted MR images of the brains from different groups were obtained, and the slice thickness was 1.0 mm. The ventricular systems were reconstructed based on T2-weighted images with 3D Slicer (4.11, USA), and the ventricular volumes were calculated.

### CSF collection and albumin content detection

CSF was collected by inserting a glass capillary through the cisterna magna. The collected CSF was centrifuged to remove any cells or tissues. The albumin content in the prepared CSF was measured with a colorimetric albumin assay kit.

### Cytokine assays

According to the manufacturer’s instructions, the concentration of ICAM-1 protein in the choroid plexus and CSF was determined with cytokine assays from Huaying-bio (Shanghai, China).

### Immunofluorescence staining and BODIPY staining

For immunofluorescence staining, the separated choroid plexus, brain sections, and cells were washed with 0.1 M PBS, treated with 0.3% Triton X-100 for 30 min, and blocked with 5% bovine serum albumin for 1 h. The prepared samples were incubated with primary antibodies overnight at 4 °C. After washing with 0.1 M PBS 3 times for 10 min, the samples were incubated with secondary antibodies for 2 h at room temperature. Finally, the nuclei were marked with DAPI or Hoechst before observation. The primary antibodies used included anti-TLR2, anti-NLRP3, anti-AE2, anti-ZO-1, anti-claudin-5, anti-PLIN3, and anti-MMP9 antibodies. BODIPY 493/503 (1 μg/ml) dissolved in DMSO was used to stain LDs. MitoTracker Red (500 nM) was used to label mitochondria. To visualize B-CSFB leakages, rats were injected with dextran (1000 mW, 24 mg/ml, 100 µL per rat) intravenously. Two hours later, anesthetized rats were perfused, and their brain tissue was sectioned for further staining. All images were collected with the confocal microscope system LSM880 (Zeiss).

### Statistical analysis

All data are expressed as the mean±SD. Each figure legend contains details of the sample sizes and replicates. For the comparison of two groups, the two-tailed unpaired Student’s *t* test was used. One-way ANOVA followed by the Bonferroni post hoc test was used to compare means among multiple groups. Statistical analysis was performed with GraphPad Prism 8 (San Diego, CA, USA). A *p* value < 0.05 was considered statistically significant.

## Results

### NLRP3 knockout alleviated posthemorrhagic hydrocephalus and neurological deficits after ICH-IVH

The inflammatory response is a common injury factor after CNS injury, including hemorrhage. Increasing evidence supports an essential role for the NLRP3 inflammasome in the acute phase of inflammatory diseases^18^. We first evaluated whether NLRP3 knockout had any apparent effect on the degree of hydrocephalus and neurological function in rats. Three days after ICH-IVH, WT and Nlrp3^−/−^ rats showed significant neurofunctional changes but no influence on basal features.

**Fig. 1:**
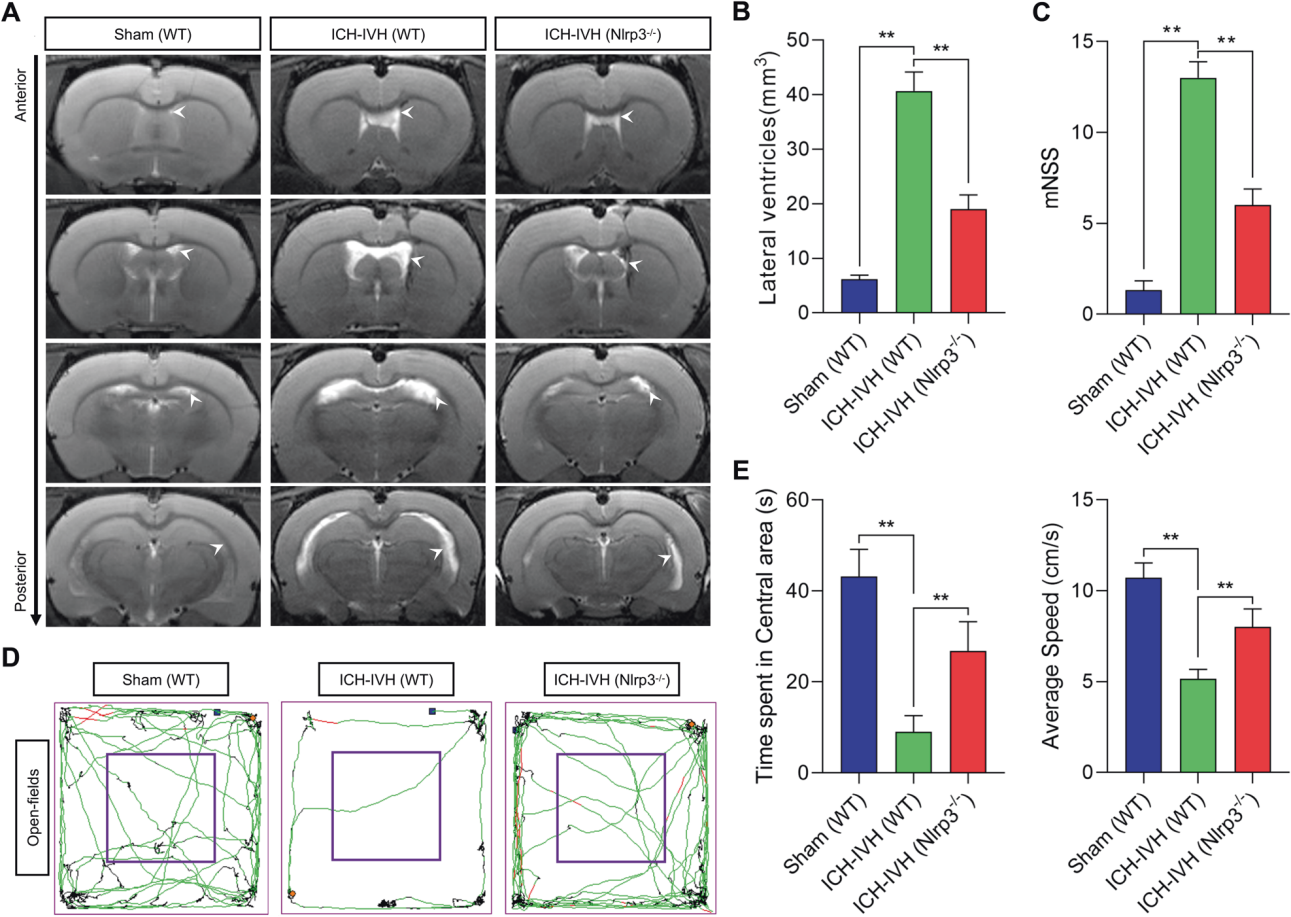
Evaluation of hydrocephalus and neurological function with or without NLRP3 knockout after ICH-IVH. **A** T2-weighted images of different groups of rats showed ventricular dilatation, and the white arrows indicate the lateral ventricles. **B** Trajectories of different groups of rats after ICH-IVH in the open-field experiment. **C** Statistical results of the central area movement time and mean velocity of different groups throughout the open-field experiment (*n* = 6, one-way ANOVA). **D** Statistical analysis of lateral ventricular volumes of different groups based on MRI images (*n* = 3, one-way ANOVA). **E** The mNSS of different groups (*n* = 6, one-way ANOVA). The results are expressed as the mean ± SD, **P < 0.01.

First, the lateral ventricular volumes were measured using T2-weighted imaging, the primary evaluation index to assess hydrocephalus (Fig. 1a). According to T2-weighted images, WT rats had more severe ventricular dilatation than Nlrp3^−/−^ rats after ICH-IVH (Fig. 1b). Next, we explored the degree of lateral ventricular expansion and whether it is related to neurological function, including motor and cognitive functions. All results indicated that rats experienced obvious neurological deficits after ICH-IVH. Motor function was assessed by the mNSS and the average movement speed on the open-field test. The results showed that Nlrp3^−/−^ rats had a higher movement speed than WT rats, and the same results were obtained on the mNSS (Fig. 1c–e). Regarding cognitive function, the time spent in the central area of the open-field test was measured to investigate whether NLRP3 knockout rescued cognitive function after ICH-IVH. Compared with WT rats, Nlrp3^−/−^ rats exhibited improved cognitive function after ICH-IVH (Fig. 1d, e). Taken together, our results showed that Nlrp3^−/−^ could enhance the outcome of hydrocephalus after hemorrhage.

### RNA-seq and proteomics analysis indicated activation of choroid plexus NLRP3 via TLR2 after ICH-IVH and tight junction disruption

**Fig. 2:**
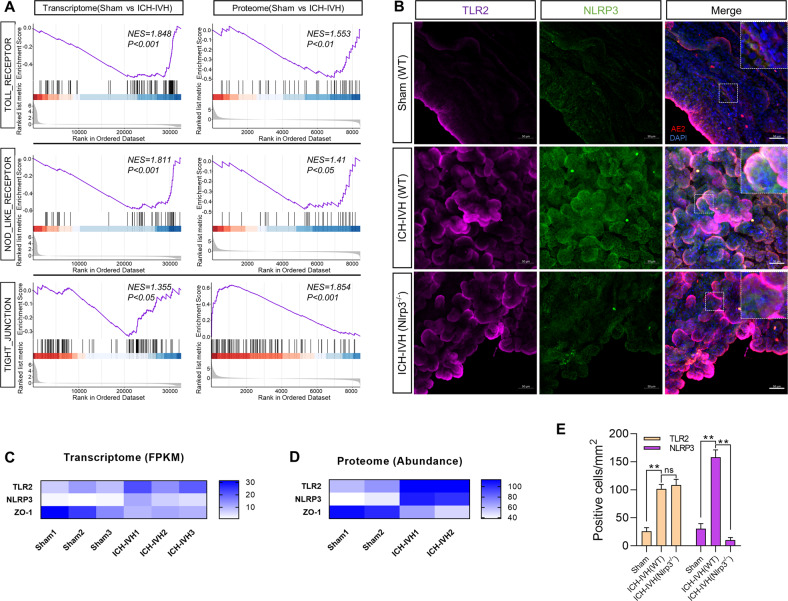
Choroid plexus sequencing results confirmed that TLR2/NLRP3 pathway activation and tight junctions were abolished at 3 days after ICH-IVH. **A** GSEA enrichment plots demonstrating the degree of correlation between genes in the Toll receptor-, NOD-like receptor-, and tight-junction-associated categories and proteins in choroid plexus tissues from the control and ICH-IVH groups. **B** Immunofluorescent staining images of TLR2 and NLRP3 in the choroid plexus. Scale bar = 50 μm. **C** Representative gene abundance heatmap of transcriptome sequencing: TLR2, NLRP3, ZO-1. **D** Heatmap of proteins screened after proteomic sequencing GSEA enrichment analysis: TLR2, NLRP3, ZO-1. **E** Analysis of TLR2- and NLRP3-positive cell counting results of choroid plexus immunofluorescence staining (*n* = 3, one-way ANOVA). The results are expressed as the mean ± SD, **P < 0.01, ns *P* > 0.05.

After ICH-IVH, blood components act directly on the choroid plexus to activate its inflammatory response. We performed a preliminary investigation by sequencing to elucidate how choroid plexus NLRP3 is activated after ICH-IVH and the effect on choroid plexus function. Based on GSEA enrichment analysis of the choroid plexus transcriptome and proteome sequencing, we found that the Toll receptor family was significantly activated after ICH-IVH (including TLR2); NOD-like receptors were also activated (including NLRP3), and tight-junction-related functions were impaired (including ZO-1) (Fig. 2a). Combined with the GSEA enrichment results, the heatmap of representatives showed pathway-related genes in the transcriptome sequencing based on fragments per kilobase million (FPKM) values and found that TLR2 and NLRP3 were upregulated in the choroid plexus after ICH-IVH; in contrast, ZO-1 showed the opposite trend (Fig. 2c). Furthermore, the heatmap of proteomes led to the same conclusion that activating the TLR2/NLRP3 pathway disrupted tight junctions (Fig. 2d). Next, we verified the expression of TLR2 and NLRP3 in the choroid plexus using immunofluorescence and found a significant increase in TLR2- and NLRP3-positive cells after ICH-IVH, while there was no effect on TLR2 expression after NLRP3 knockout, suggesting that TLR2 may be an upstream molecule of NLRP3 (Fig. 2b, e). These results demonstrated the activation of NLRP3 in the choroid plexus after ICH-IVH and the possibility that TLR2 is an upstream signaling molecule of NLRP3. In particular, the disruption of tight junctions in the choroid plexus was found after ICH-IVH, implying impaired B-CSFB function, which may be a potential pathological mechanism of PHH.

### NLRP3 knockout protected B-CSFB function after ICH-IVH

The CNS is tightly sealed from the changeable milieu of the blood by a B-CSFB consisting of the choroid plexus. It has been demonstrated that increased inflammation affects B-CSFB integrity in AD pathology^19^. Since Nlrp3^−/−^ rats showed improved hydrocephalus and neurological deficits, we therefore examined whether the permeability of the B-CSFB changed after PHH with NLRP3 knockout. It is well known that tight junctions between choroid plexus epithelial cells are the main intercellular junctions that maintain the integrity of the B-CSFB. Thus, we next analyzed the B-CSFB integrity of WT and Nlrp3^−/−^ rats by various methods.

**Fig. 3:**
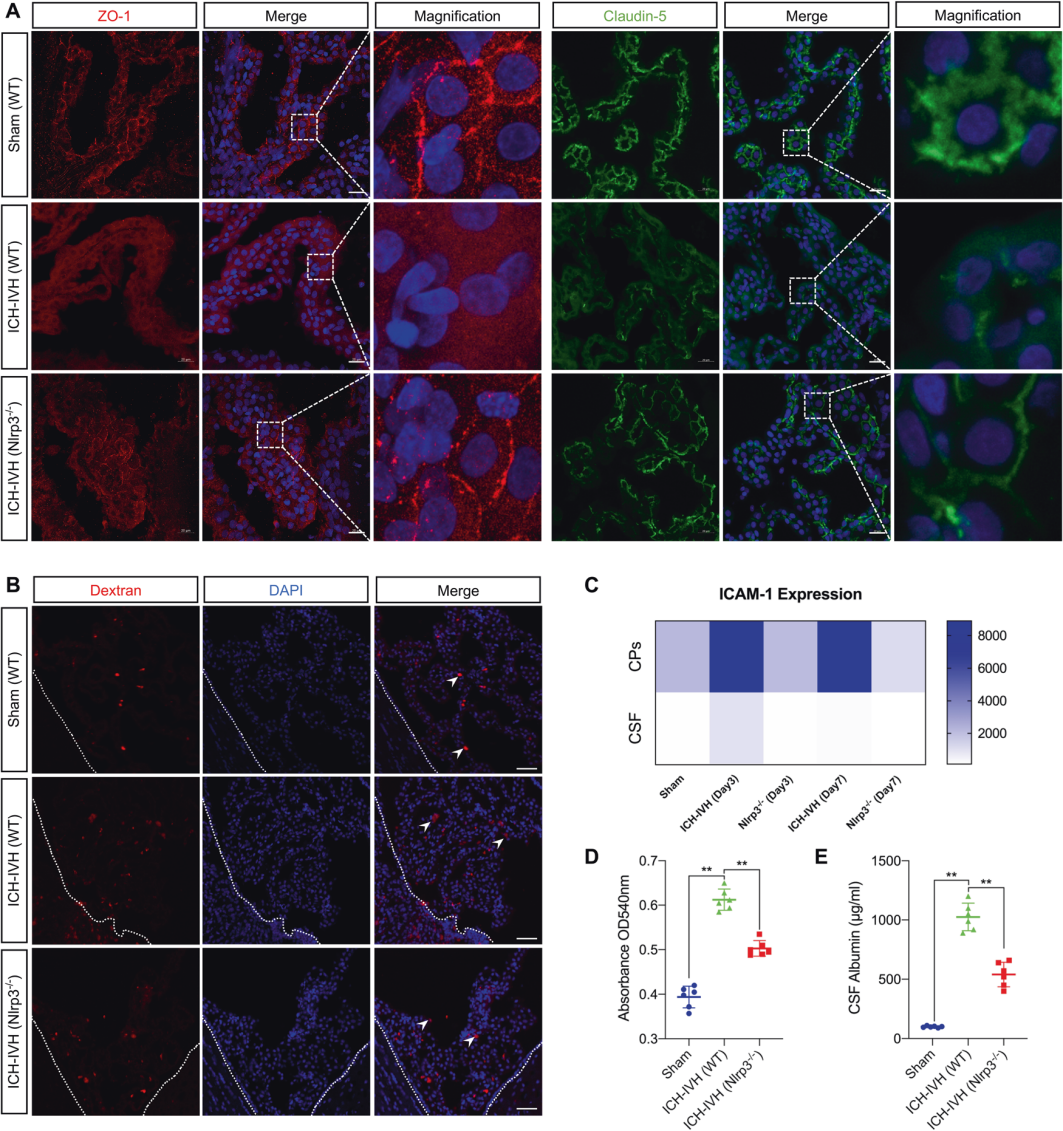
Assessment of B-CSFB integrity in WT rats and Nlrp3^−/−^ rats 3 days after ICH-IVH. **A** Representative images of immunofluorescence staining for ZO-1 and claudin-5 in the choroid plexus of different groups. Scale bar = 20 μm. **B** Pictures of B-CSFB leakage assessed by dextran. Scale bar = 50 μm. **C** Heatmap of ICAM-1 expression in the choroid plexus and CSF (*n* = 3, one-way ANOVA). **D** Results of the absorbance assay of CAFs in different groups after dextran injection (*n* = 3, one-way ANOVA). **E** Statistical results of CSF albumin content in different groups (*n* = 6, one-way ANOVA). The results are expressed as the mean ± SD, **P < 0.01.

We examined changes in tight junction proteins in the choroid plexus 3 days after ICH-IVH. The immunostaining results of ZO-1 and claudin-5 in the choroid plexus showed that Nlrp3^−/−^ improved the integrity of the B-CSFB compared with the WT rats after ICH-IVH. Interestingly, the destruction of the tight junction was mainly located on the luminal side, not the basal side, which points to selective disruption and functional response (Fig. 3a). Additionally, dextran (1000 mW) was used to evaluate the integrity of the B-CSFB, and we found that Nlrp3^−/−^ reduced the leakage of dextran compared with WT rats (Fig. 3b, d). The CSF was also a target to indirectly reflect the function of the B-CSFB. We used cytokine kits to measure the contents of ICAM-1 in the choroid plexus and CSF and found that Nlrp3^−/−^ rats had a higher expression level than WT rats after ICH-IVH (Fig. 3c). The B-CSFB separates blood from CSF, and there are significant differences in the expression fraction of various proteins in the two fluids under normal conditions, most notably in the amount of albumin^20^; so we evaluated the changes in albumin in the CSF of different groups of rats and found a significant increase in CSF albumin levels after ICH-IVH, which was reversed by Nlrp3^−/−^ (Fig. 3e). This evidence again supports the idea that NLRP3 knockout can repair the disruption of the B-CSFB after ICH-IVH, which may be one of the important injury targets for the development of PHH.

### Quantitative transcriptomic and proteomic analysis of the choroid plexus revealed functional changes in lipid metabolism that reduced the abundance of LDs in Nlrp3^−/−^ rats

**Fig. 4:**
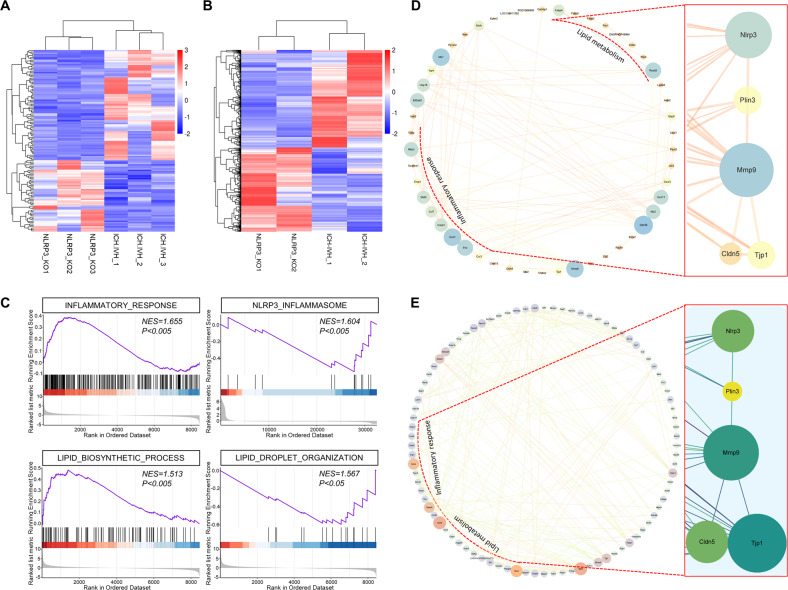
Bioinformatic analysis revealed an inflammatory response and lipid metabolism changes in Nlrp3^−/−^ rats 3 days after ICH-IVH. **A** Heatmap of differentially expressed genes from transcriptome sequencing of Nlrp3^−/−^ rats versus WT rats. **B** Heatmap of differentially expressed proteins from proteome sequencing. **C** GSEA of sequencing results showing a diminished inflammatory response and altered lipid metabolism in Nlrp3^−/−^ rats. **D** Network images showing inflammatory, lipid-related, and potentially related genes after ICH-IVH with or without NLRP3 knockout. **E** Protein–protein interaction network of inflammatory and lipid-related proteins.

To investigate the potential mechanism by which NLRP3 knockout could improve PHH by affecting B-CSFB function, we performed transcriptome and proteome sequencing analysis of choroid plexus tissues from WT and Nlrp3^−/−^ rats 3 days after ICH-IVH. The results of both transcriptome and proteome sequencing showed that the expression of several genes and proteins was upregulated or downregulated between the two groups (transcriptome: fold change ≥2 as the threshold of change, *P* ≤ 0.05 as the differentially expressed gene screening criterion; proteome: fold change ≥1.2 as the threshold of change, *P* ≤ 0.05 as the differentially abundant protein screening criterion). Overall, 111 genes were upregulated and 149 genes were downregulated in the transcriptome (Fig. 4a), while 516 proteins were upregulated and 445 proteins were downregulated in the proteome (ICH-IVH(WT) versus ICH-IVH(Nlrp3^−/−^)) (Fig. 4b). Heatmaps based on differentially expressed genes or proteins were used to visualize these results clearly. Furthermore, we analyzed the functions of these differentially expressed genes and proteins by GSEA and found that lipid metabolism functions were altered after NLRP3 knockout. Specifically, LD formation was dysfunctional. PLIN3 is a key gene that regulates LD formation, and its abundance reflects the status of the process. Additionally, NLRP3 knockout attenuated the inflammatory response, most notably the expression of NLRP3 (Fig. 4c). Since NLRP3 can regulate the PLIN3 level, whether LDs influence the permeability of B-CSFBs needs further investigation. By targeting NLRP3 and PLIN3 and combining the transcriptome and proteome sequencing results of differentially expressed genes and proteins, we used network planning to predict possible pathways (Supplementary Tables 1–2). Joint analysis of the transcriptome and proteome sequencing data suggested that LDs act on tight junctions of the B-CSFB (Fig. 4d, e).

In conclusion, we demonstrated that altered B-CSFB function after NLRP3 knockout is accompanied by lipid homeostasis imbalance, especially with regard to LD formation, which may be a potential pathway of NLRP3.

### Inhibition of LD formation improves the B-CSFB and the outcome of PHH

**Fig. 5:**
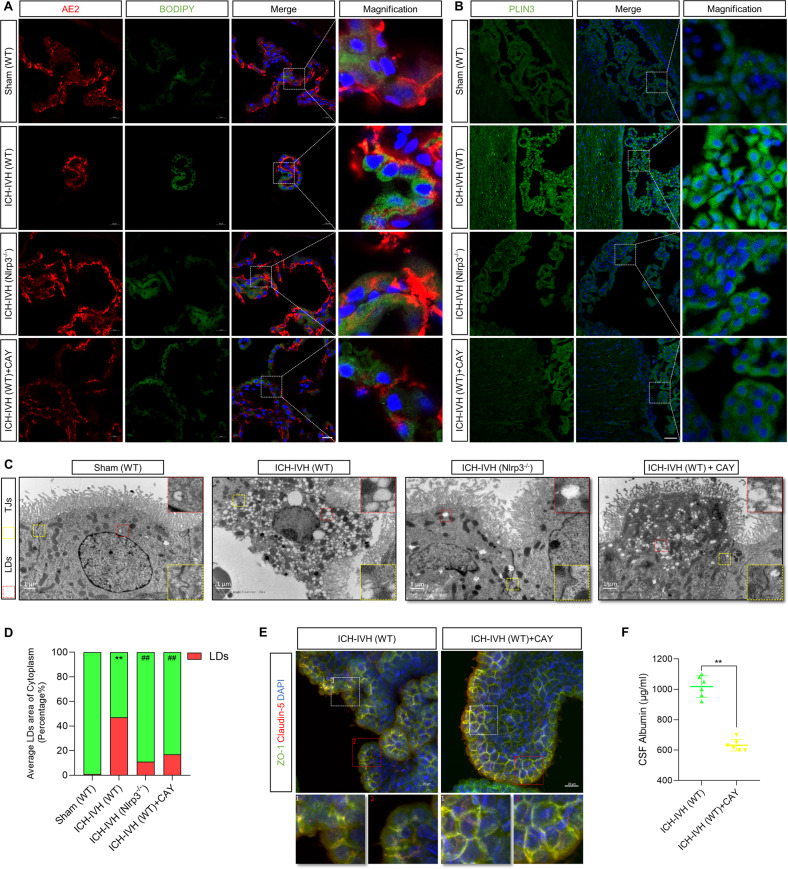
Inhibiting LD formation improved B-CSFB permeability and neurological deficits after ICH-IVH. **A** BODIPY staining of LDs in the choroid plexus (marker with AE2). Scale bar = 20 μm. **B** Representative images of PLIN3 staining indicating LDs in the choroid plexus. Scale bar = 50 μm. **C** TEM images showed tight junctions and LDs in the choroid plexus. Scale bar = 1 μm. **D** LD area percentage of total cytoplasm according to TEM images. **P < 0.01, sham (WT) versus ICH-IVH (WT); ##P < 0.01, ICH-IVH (WT) versus others (*n* = 3, one-way ANOVA). **E** Immunofluorescence staining of ZO-1 and claudin-5 in the choroid plexus after inhibiting LDs. Scale bar = 20 μm. **F** CSF albumin content after ICH-IVH with or without CAY10650 treatment (*n* = 6, unpaired *t* test). The results are expressed as the mean ± SD, **P < 0.01.

Having previously demonstrated that NLRP3 knockout affects LD formation, we hypothesized that it might be one of the critical pathways involving the tight junctions of the B-CSFB based on sequencing results, which we next verified by multiple methods. First, we labeled and quantified LDs in the choroid plexus with BODIPY LD dye and PLIN3 (an essential protein for LD formation) and found that LD formation in the choroid plexus increased after ICH-IVH. Nlrp3^−/−^ rats exhibited decreased LD formation compared with the WT ICH-IVH group. Then, we selected CAY10650, a specific inhibitor of PLIN3, to intervene and found that it reduced LD formation in the choroid plexus (Fig. 5a, b). To visualize and evaluate LDs, which are considered an intracellular organelle closely related to the inflammatory response, we performed a quantitative analysis of LDs in different groups using TEM and evaluated the area of LDs in choroid plexus epithelial cells of different groups based on the area of LDs in the true cytoplasm and came to the same conclusion as with BODIPY and PLIN3 staining (Fig. 5c, d).

NLRP3 knockout improved B-CSFB tight junctions, and PLIN3, which is the main protein that adjusts LD formation, was regulated by NLRP3 after ICH-IVH. Next, we observed the expression of the tight junction-related proteins ZO-1 and claudin-5 after inhibiting PLIN3 using CAY10650 and found that interfering with LD formation improved choroid plexus tight junctions (Fig. 5e). Additionally, we detected albumin in CSF and found that inhibiting LD formation decreased the abundance of albumin after ICH-IVH (Fig. 5f).

**Fig. 6:**
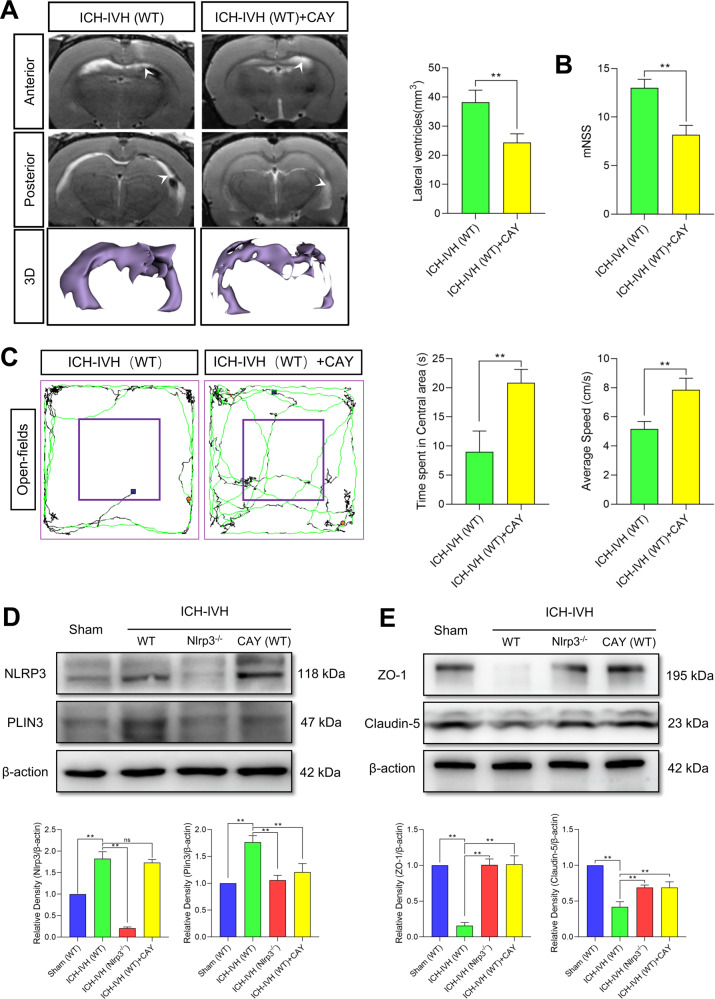
NLRP3 mediates tight junction dysfunction by altering LD formation. **A** T2-weighted and 3D reconstruction images showed lateral ventricles and volume analysis results (*n* = 3, unpaired t test). **B** Statistical analysis of the mNSS results (*n* = 6, unpaired *t* test). **C** Trajectory images and analysis results of the open-field tests with and without CAY10650 treatment after ICH-IVH (*n* = 6, unpaired t test). **D** Western blot detection of NLRP3 and PLIN3 in the choroid plexus and quantified abundance results (*n* = 3, 6 rats per sample, one-way ANOVA). **E** Western blot images and statistical results showing the expression of the tight-junction-related proteins ZO-1 and claudin-5 in the choroid plexus (*n* = 3, 6 rats per sample, one-way ANOVA). The results are expressed as the mean ± SD, **P < 0.01.

Since directly interfering with LD formation can improve B-CSFB function, it is meaningful to observe whether the neurological function and hydrocephalus of rats are improved as well. According to T2-weighted images and 3D reconstruction images of ventricles, inhibiting LD formation decreased lateral ventricle dilation after ICH-IVH (Fig. 6a). Finally, we assessed neurological function in the open-field test and with the mNSS and found that CAY10650 treatment improved neurological function after PHH (Fig. 6b, c).

Finally, we quantified the expression of the related proteins NLRP3, PLIN3, ZO-1, and claudin-5 in different groups by WB and confirmed that there was no effect on NLRP3 expression when LD formation was inhibited by CAY10650. NLRP3-regulated LD formation (Fig. 6d); however, it also increased the expression of ZO-1 and claudin-5, thus strengthening tight junctions (Fig. 6e).

In summary, LDs were synthesized en masse in the choroid plexus after ICH-IVH, and NLRP3 could regulate the formation of LDs. In addition, intracellular LDs had a regulatory effect on the tight junctions of the B-CSFB.

### LDs adjusted B-CSFB integrity by influencing mitochondrial function

Herein, we found that LDs interacting with mitochondria released mitochondrial reactive oxygen species (ROS) and induced mitochondrial membrane potential (MMP) depolarization. Additionally, the mitochondria swelled, and cristae were reduced after ICH-IVH in the choroid plexus (Supplementary Fig. 1a).

First, the relationship between LDs and mitochondria was assessed by TEM, which proved that LDs functioned by influencing mitochondrial function (Supplementary Fig. 1b–h). After ICH-IVH, LDs formed and bound to mitochondria (Supplementary Fig. 1b). Via TEM, we observed the disappearance of the mitochondrial cristae and shrinkage of the membranes after ICH-IVH, but NLRP3 knockout or CAY10650 treatment inhibited these changes (Supplementary Fig. 1c, f). Next, we evaluated mitochondrial function, mainly the alteration in the MMP and the evolution of mitochondrial ROS. The MMP changes in response to mitochondrial function and stress under different conditions. JC-1 was used to detect the MMP of the choroid plexus, and MMP depolarization was found to occur after ICH-IVH. NLRP3 knockout or LD formation inhibition using CAY10650 restored the MMP and protected mitochondrial function (Supplementary Fig. 1d, g). Next, we assessed mitochondrial ROS changes with MitoSOX Red and found that mitochondrial ROS production was significantly increased after ICH-IVH (Supplementary Fig. 1e, h). However, NLRP3 knockout or CAY10650 treatment reduced mitochondrial ROS production to play a protective role. Overall, the interaction of LDs with mitochondria influenced mitochondrial function, which contributed to B-CSFB dysfunction by increasing the release of mitochondrial ROS after ICH-IVH.

### Clearing mitochondrial ROS with MitoQ improved the function of the B-CSFB by downregulating MMP9 after ICH-IVH

LDs interacting with mitochondrial release increased the amounts of mitochondrial ROS to influence the permeability of the B-CSFB after ICH-IVH. To determine the intermediate molecules of mitochondrial ROS that affect tight junctions in the choroid plexus, MMP9 was selected as a bridge, and MMP9 data were combined with the sequencing results.

MMP9 plays a particularly prominent role in the matrix metalloprotease family, which can respond to mitochondrial ROS stimulation. First, we examined the expression of MMP9, which was highly colocalized with the tight-junction-related protein ZO-1, in the choroid plexus using immunofluorescence. After ICH-IVH, MMP9 activation was significantly accompanied by disruption of tight junctions. After CAY10650 was used to inhibit LD formation or MitoQ was used to remove mitochondrial ROS, improvements in tight junctions were observed, with a concomitant decrease in MMP9 expression in the choroid plexus (Supplementary Fig. 2a). To further confirm this finding, we performed a quantitative analysis of MMP9 and ZO-1 expression using Western blotting and found that MMP9 expression was upregulated and that ZO-1 expression was downregulated after ICH-IVH. Regardless of whether PLIN3 was inhibited or mitochondrial ROS were cleared, both methods protected tight junctions in the choroid plexus through MMP9 (Supplementary Fig. 2b). Interestingly, NLRP3 expression was inhibited by MitoQ treatment, which is consistent with the findings of other studies showing that ROS can activate NLRP3 through the intracellular pathway. This process forms positive feedback to aggravate B-CSFB damage. These findings proved that LDs mediated mitochondrial ROS release and destroyed the B-CSFB through MMP9 after ICH-IVH.

### NLRP3 activation in epithelial cells resulted in LD formation and permutation of tight junctions

We then investigated whether NLRP3 activation in primary CPECs mediated LD formation and influenced tight junctions between CPECs in vitro. Lipopolysaccharide (LPS) is a common endotoxin from gram-negative bacteria that has been proven to activate the inflammatory response (including the NLRP3 inflammasome) in various cells, including macrophages, endothelial cells, and epithelial cells^21^.

**Fig. 7:**
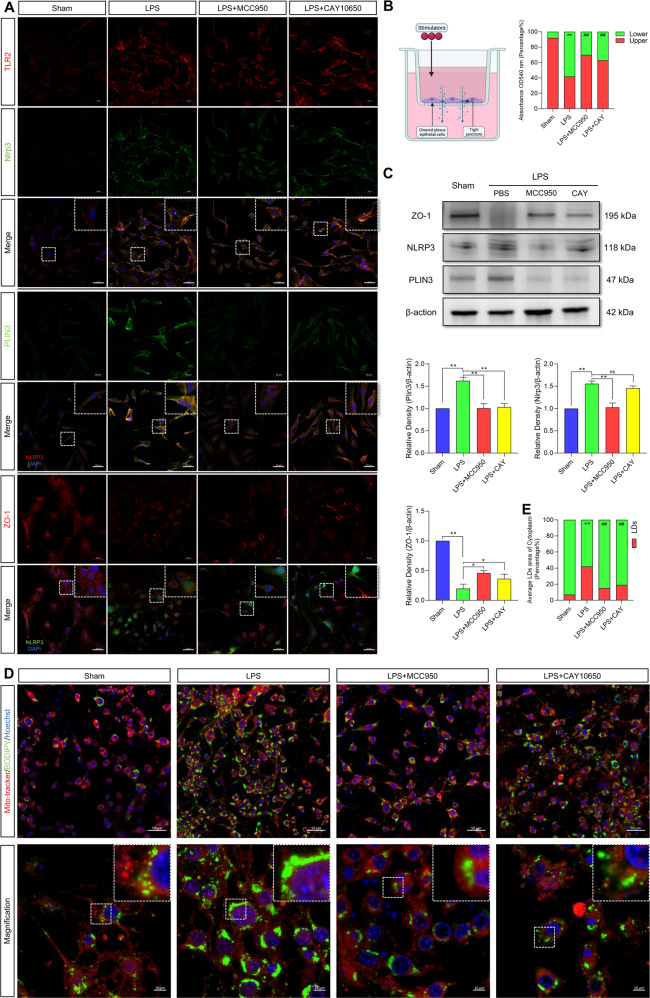
LPS induces NLRP3 activation in CPECs and disrupts tight junctions by regulating LD formation. **A** Immunofluorescence multiple-localization staining results of TLR2, NLRP3, PLIN3 and ZO-1 in CPECs under LPS stimulation and MCC950 or CAY10650 treatment. Scale bar = 50 μm. **B** Barrier function assessment in a CPEC-composed B-CSFB as measured by dextran leakage (*n* =3, one-way ANOVA). **C** The expression of ZO-1, NLRP3 and PLIN3 in LPS-induced CPECs and MCC950 or CAY10650 treatment (*n* = 3, one-way ANOVA). **D** Images of LDs stained with BODIPY and mitochondria labeled with MitoTracker. Scale bar = 50 μm (upper), Scale bar = 10 μm (lower). **E** Results of LD coverage area of cells (*n* = 3, one-way ANOVA). The results are expressed as the mean ± SD, **P < 0.01, *P < 0.05, ns *P* > 0.05.

LPS was used to induce NLRP3 activation in CPECs; MCC950 was used to inhibit NLRP3 activation, and CAY10650 was used to inhibit PLIN3 to interfere with LD formation. By immunofluorescence staining, we observed that the activation of NLRP3 and PLIN3 after LPS induction was accompanied by the disruption of tight junctions (ZO-1 expression decreased) in CPECs and found that inhibiting NLRP3 decreased LD formation and protected tight junctions. Inhibiting PLIN3 improved tight junctions but did not influence NLRP3 expression (Fig. 7a). Furthermore, the constructed CPEC structure was used to simulate the B-CSFB in vivo. The content of dextran in the separated medium (upper and lower) was used to assess tight junctions. LPS treatment destroyed the tight junctions in CPECs, and inhibition of either NLRP3 or PLIN3 decreased dextran leakage by improving tight junctions (Fig. 7b). Western blotting was used to prove previous results and showed that NLRP3 and PLIN3 had higher expression levels after LPS stimulation. Inhibiting NLRP3 decreased PLIN3 expression levels and improved tight junctions. CAY10650 usage proved that PLIN3 could be regulated by NLRP3 (Fig. 7c).

LDs function by binding to multiple organelles, especially mitochondria. It has been proven that LDs interacting with mitochondria influence mitochondrial processes^10^. Mitochondria are vital for the maturation and maintenance of blood‒brain barrier (BBB) integrity by supplying ATP to endothelial cells^22^. Validating the interaction of LDs and mitochondria, LDs labeled with BODIPY were close to mitochondria stained with MitoTracker in spatial location (Fig. 7d), which provided the foundation for functional interaction. The LD contents after different treatments were also assessed by BODIPY staining, which showed the same results as before (Fig. 7e). Briefly, NLRP3-mediated LD formation to influence tight junctions in CPECs, as shown by the in vivo experimental results. Additionally, LDs destroyed tight junctions by interacting with mitochondria.

### LDs influenced mitochondrial function to adjust tight junctions

The previous in vivo experiments demonstrated that LDs disrupt tight junctions in the B-CSFB by binding to mitochondria, affecting mitochondrial ROS production through MMP9. To further verify this conclusion, we evaluated mitochondrial function and tight junctions in CPECs. First, we found that MMP depolarization and mitochondrial ROS production increased after LPS-induced NLRP3 activation; then, we inhibited NLRP3 with MCC950 and confirmed that the MMP recovered and that mitochondrial ROS production was reduced compared with that after LPS induction alone. We also used MitoQ, which has been proven to be an effective mitochondrial ROS scavenger, and found that it improved mitochondrial function and significantly reduced mitochondrial ROS production (Supplementary Fig. 3a, b, d, e).

Since mitochondrial ROS regulate tight junctions by affecting MMP9 levels, we next quantified the expression of MMP9 and ZO-1 in different treatment groups by Western blotting and found that either inhibition of NLRP3 or removal of mitochondrial ROS attenuated the high expression of MMP9 after LPS induction while upregulating ZO-1 expression (Supplementary Fig. 3c). These results confirmed that LDs mediated mitochondrial ROS production to destroy tight junctions through MMP9 and that scavenging of mitochondrial ROS by MitoQ produced a protective effect.

## Discussion

Our experiments showed that NLRP3 knockout improved B-CSFB function after ICH-IVH. NLRP3 located in the choroid plexus was activated after ICH-IVH in WT and Nlrp3^−/−^ ICH-IVH rat models and in in vitro CPEC culture. Next, we proved that NLRP3 regulates LD formation by affecting PLIN3 expression, and the synthesized LDs interacting with mitochondria affect mitochondrial function to produce more mitochondrial ROS, which disrupts the tight junctions in the B-CSFB through MMP9; this view was verified again using in vitro cellular experiments.

The choroid plexus, as the B-CSFB within the ventricles, bears the brunt of environmental stress and disruption after hemorrhage; however, its functional and pathological roles remain to be clarified^23^. The B-CSFB constituted by the choroid plexus plays an important role in CNS disorders, whether genetic developmental diseases from infancy or a variety of neurodegenerative diseases with a high incidence of disruption of the B-CSFB. In the case of developmental disorders, a variety of congenital neurological diseases are caused by multiple genetic deletions in the choroid plexus and can cause severe hydrocephalus or other problems^24,25^; furthermore, in the pathological process of AD, studies have confirmed that B-CSFB function gradually decreases with age, and improving B-CSFB function can reduce the content of Aβ or tau proteins in CSF^26^. The B-CSFB regulates the transport of substances on both sides of the barrier, acts as an immune barrier, and provides adhesion sites for immune cells, which support the recruitment of these cells after injury. Choroidal plexus immune cells primarily include macrophages and microglia, and the recruited immune cells affect choroidal plexus function^27,28^. All evidence suggests that B-CSFB disruption allows some components within blood and brain tissue to enter the CSF. In contrast, the differences in the presence of multiple elements in blood and CSF provide opportunities for the transport of cells, proteins, ions, and water molecules into the CSF after barrier disruption. Regarding B-CSFB, we hypothesized that in the PHH rat model, blood components in the CSF and immune cells stimulate the choroid plexus, leading to inflammatory response activation. Therefore, we verified the inflammatory effect on B-CSFB function and confirmed that the disruption of the B-CSFB is partly mediated by the inflammatory response.

The NLRP3 inflammasome, as one of the critical molecules of the immune and inflammatory response, involves complex and diverse pathways and functions that are activated in various pathological processes or after LPS-induced inflammation. Our previous studies have proven that NLRP3 is activated in the choroid plexus after ICH-IVH in the acute phase. It has been demonstrated that Toll-like receptor activation in the choroid plexus after IVH is involved in the pathology of hydrocephalus. In addition, there is extensive evidence that Toll-like receptors can activate NLRP3, especially TLR4^29,30^. Based on choroid plexus transcriptome and proteome sequencing data, our studies demonstrated that NLRP3 could be activated after ICH-IVH through TLR2 in the choroid plexus. NLRP3 activation causes different degrees of cellular damage, ranging from cellular dysfunction and intercellular communication impairment to cellular scorching^29^; how does NLRP3 disrupt the B-CSFB in the choroid plexus? Combined with the sequencing results, we also found alterations in lipid metabolism function, especially LD formation. We found that many LDs were synthesized in the choroid plexus after ICH-IVH. LD formation was reduced after knockout of NLRP3, a phenomenon consistent with NLRP3 and LD formation demonstrated in hepatocytes^31^. However, there is still some controversy about the relationship between NLRP3 and LDs; the traditional view is that intracellular LD formation is mainly regulated by the endoplasmic reticulum and is increased under conditions of endoplasmic reticulum stress^32^; however, some studies on the relationship between NLRP3 and endoplasmic reticulum stress support the idea that endoplasmic reticulum stress can activate NLRP3^11,33,34^, and these conclusions appear to be at odds. This may be because NLRP3 may not regulate LD formation through the endoplasmic reticulum pathway^35,36^. Based on related studies and our experimental findings, we speculate that NLRP3-mediated LD formation in the choroid plexus does not occur via the endoplasmic reticulum pathway but may be stimulated directly by other organelles, such as the Golgi apparatus or a downstream molecule, after NLRP3 activation, and the exact path needs to be confirmed by further investigation.

Since NLRP3 can adjust LD formation in the choroid plexus, is there an effect of NLRP3-regulated LD formation on B-CSFB tight junctions under injury conditions? LDs can bind to organelles such as mitochondria or lysosomes to participate in the regulation of cellular homeostasis^37^, and their regulation of mitochondria mainly affects the level of oxidative stress, especially the level of mitochondrial ROS. Of course, LD binding to mitochondria also has a regulatory effect on mitochondrial autophagy^38,39^. The other major area of study is their interaction with lysosomes, which focuses on their impact on cellular autophagy and the maintenance of internal environmental stability in response to changes in the external environment^40^. As an intermediate organelle in the regulation of the B-CSFB by LDs, mitochondria are significantly dysfunctional in a variety of disease conditions. For example, some pathological states involve mitochondrial membrane depolarization that renders these organelles nonfunctional; additionally, given that mitochondria are the main organellar sources of ROS, mitochondrial dysfunction can cause cellular oxidative/antioxidative imbalance^41,42^. It has been demonstrated that ROS are involved in secondary injury after ICH, especially in highly oxygen-depleted neuronal cells^43^. In addition, in the choroid plexus, which contains epithelial cells with highly secretory properties, we found that interfering with LD formation could inhibit the production of mitochondrial ROS, while it has been demonstrated that ROS can activate NLRP3^44^.

The relationship between LDs and mitochondria is still unclear. Accumulated studies have revealed that physical contact between LDs and mitochondria is important for their functions^38,45^. Physical contact provides a possibility for functional interaction. One model of interaction is the “kiss-and-run” model, with mitochondrial motility adjusting mitochondrial fusion with LDs, which provides a mechanism of adherence to LDs. Some of these mitochondria are defined as peridroplet mitochondria (PDM), and their function is different from that of cytoplasmic mitochondria (CM). The unbalanced proportions of PDM and CM causing dysfunction remain to be explored in pathological conditions^45,46^. Another form of contact, termed anchoring, has also been found; mitochondria that are engaged in this form of contact are known as LD-anchored mitochondria (LDAM) and are located in oxidative tissue. Several proteins were found to mediate this contact, especially PLINs^38,39^. Both contact patterns have been proven to exhibit functional interactions. In addition, there are different models for the interaction between LDs and mitochondria, and two roles for mitochondrial association with LDs have been proposed: mitochondria with a high capacity to synthesize ATP support LD expansion, and fatty acids in LDs are transported to mitochondria, feeding them^47^. Then, in the present study, exogenous stimuli (especially blood components and metabolites) acting on the choroid plexus activated NLRP3, while NLRP3 affected mitochondrial ROS production by regulating LD formation, and the generated mitochondrial ROS counteracted NLRP3, producing a cascade amplification effect and aggravating the injury. We observed the physical contact of LDs and mitochondria and then obtained the conclusion above. However, the process and mechanism by which LDs influence mitochondria are still unclear and need further study.

Of course, there are still some shortcomings in our study. First, we proposed that NLRP3 can regulate LD formation, and it is confirmed by various lines of evidence that NLRP3 can indeed regulate LD formation; however, NLRP3 is a complex (containing ASC and Caspase-1), and it can shear pro-IL-1β and pro-IL-18 and then release IL-1β and IL-18. More experiments are needed to clarify which component promotes LD formation and related pathways; in addition, the detailed mechanisms of choroidal NLRP3 activation need further investigation. Second, we clarified that LDs act by interacting with mitochondria to affect mitochondrial ROS production and confirmed this idea by inhibiting LD formation. Although the interaction between LDs and mitochondria has been reported in many studies, the process by which the two act through the “kiss-and-run” model or some anchoring proteins that cause mitochondrial damage remains to be explored. Finally, our intervention methods, including both NLRP3 knockout and inhibitors, do not exclusively target the choroid plexus; more effective methods need to be explored. The present study demonstrates the importance of the B-CSFB in the pathological process of PHH and identifies several new targets for intervention, including NLRP3, LDs, and mitochondrial ROS, toward which we can aim new therapeutic approaches to improve the treatment of patients with PHH.

**Fig. 8:**
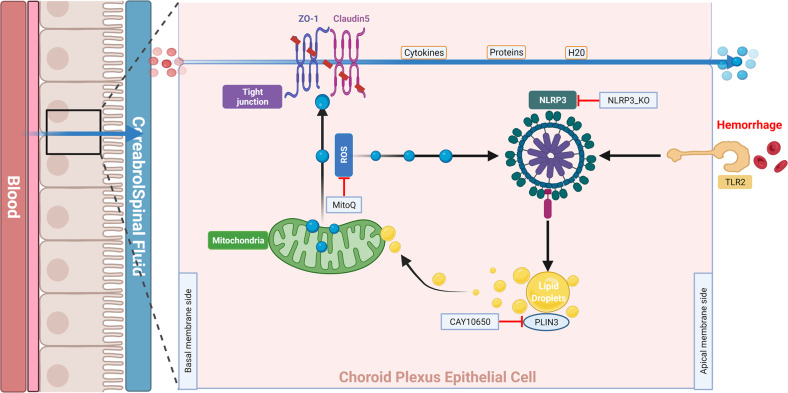
Schematic representation of the mechanism. After ICH-IVH, NLRP3 was activated in the choroid plexus by TLR2. Next, NLRP3-mediated LD formation, and LDs combined with mitochondria to influence their function and mitochondrial ROS production. Mitochondrial ROS destroyed the B-CSFB through MMP9.

In summary, we are the first to show that NLRP3-mediated dysfunction of the B-CSFB accelerated neurological deficits and hydrocephalus, at least in part, through the formation of LDs in the choroid plexus, which then interacted with mitochondria and increased mitochondrial ROS release to destroy tight junctions in the choroid plexus after ICH-IVH (Fig. 8). Thus, novel strategies designed to protect the B-CSFB may provide an effective therapeutic approach for PHH.

## Supplementary information

Supplementary Materials

Supplementary Tables

## Data Availability

All data generated or analyzed during this study are included in this published article and supplementary materials. The datasets used and/or analyzed during the current study are available from the corresponding author upon reasonable request.
