# Supplementary material for: NLRP3-dependent lipid droplet formation contributes to posthemorrhagic hydrocephalus by increasing the permeability of the blood–cerebrospinal fluid barrier in the choroid plexus
Source: Exp Mol Med. 2023 Mar 3;55(3):574–86. doi: 10.1038/s12276-023-00955-9 (PMC10073156; doi:10.1038/s12276-023-00955-9)
Supplement: Supplementary file 1 — Supplementary Materials [file 12276_2023_955_MOESM1_ESM.pdf]

Supplementary Materials

Supplementary Methods

Experiments design and grouping:

The detail of the animals used was shown as follow:

Experiment 1: Hydrocephalus and neurofunction assessment

Tests	Sham (WT)	ICH-IVH (WT)	ICH-IVH ($Nlrp3^{-/-}$)	ICH-IVH (WT)+CAY10650
MRI	3	3	3	3
Open-fields	6	6	6	6
mNSS	6	6	6	6

(Notes: Number of rats; Time-point: 3 days; Total number: 24)

Experiment 2: Transcriptome and proteome sequencing

Tests	Sham (WT)	ICH-IVH (WT)	ICH-IVH ($Nlrp3^{-/-}$)
Transcriptome	18	18	18
Proteome	12	12	12

(Notes: Number of rats; Time-point: 3 days; Total number: 90)

Experiment 3: Immunofluorescence staining (IF), Western blots (WB), and Transmission electron microscopy (TEM)

Tests	Sham (WT)	ICH-IVH (WT)	ICH-IVH ($Nlrp3^{-/-}$)	ICH-IVH (WT)+CAY10650	ICH-IVH (WT)+MitoQ
IF	3	3	3	3	3

WB	18	18	18	18	18
TEM	3	3	3	3	-

(Notes: Number of rats; Time-point: 3 days; Total number: 117, 24 rats among of them from experiment 1 (hydrocephalus and neurofunction assessment).)

Experiment 4: CSF analysis

Tests	Sham (WT)	ICH-IVH (WT), Day3	ICH-IVH ($Nlrp3^{-/-}$), Day3	ICH-IVH (WT), Day7	ICH-IVH ($Nlrp3^{-/-}$), Day7
Dextran content (CSF)	3	3	3	-	-
Albumin (CSF)	6	6	6	6	-
ICAM-1 (CSF)	3	3	3	3	3

(Notes: Number of rats; Time-point: 3 days; Total number: 48)

Experiment 5: Mitochondrial function assessment

Tests	Sham (WT)	ICH-IVH (WT)	ICH-IVH ($Nlrp3^{-/-}$)	ICH-IVH (WT)+CAY10650
JC-1	3	3	3	3
Mito-SOX	3	3	3	3

(Notes: Number of rats; Time-point: 3 days; Total number: 24)

Experiment 6: Cell experiment

Tests	Sham	LPS	LPS+MCC950	LPS+CAY10650	LPS+MitoQ
IF, Dextran leakage	6	6	6	6	-

WB	18	18	18	18	18
JC-1, Mito-SOX, BODIPY/Mitotracker	3	3	3	3	3

(Notes: Number of rats; Time-point: 3 days; Total number: 129)

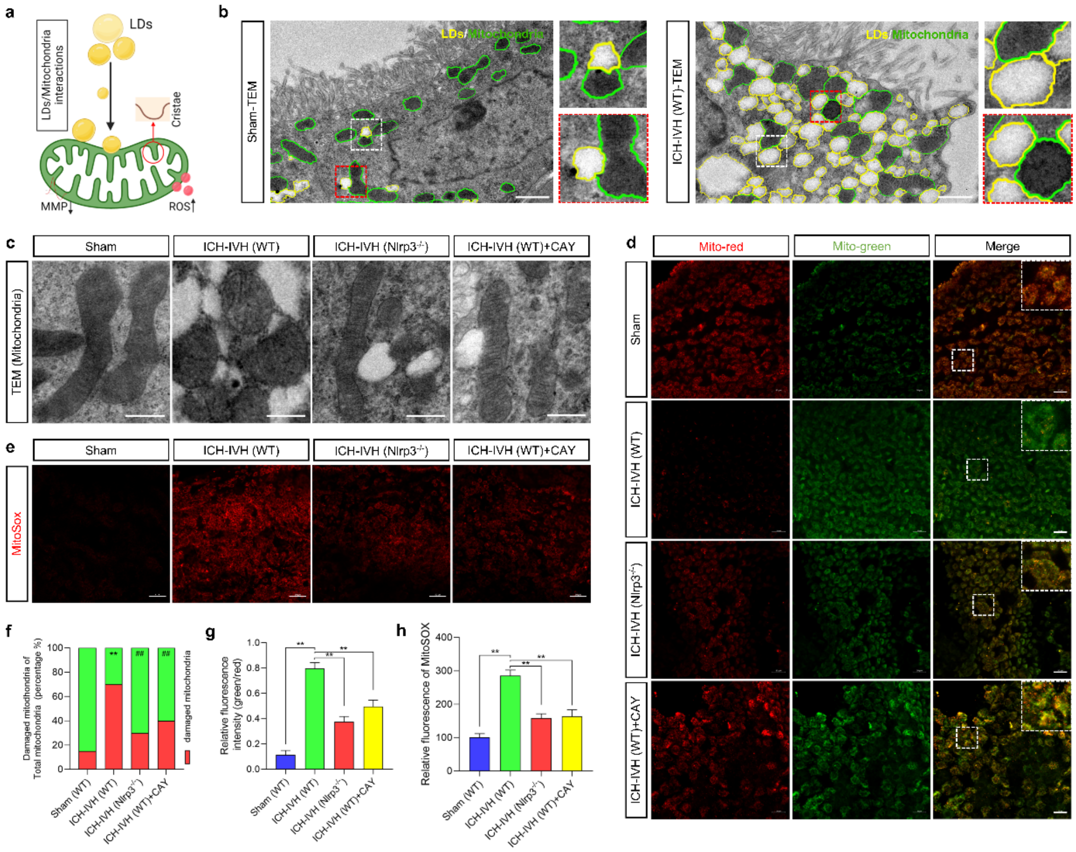

Supplementary Fig. 1. Lipid droplet accumulation influences mitochondrial function by releasing more mitochondrial ROS after ICH-IVH in the choroid plexus.

a: Schematic diagram of the hypothesis that LDs combine with mitochondria. b: TEM images of the LDs interacting with mitochondria in the choroid plexus. c: With TEM, the mitochondrial morphology changes after ICH-IVH and NLRP3 knockout or

CAY10650 treatment. Bar=0.25 μm. d: JC-1 staining images showed MMP changes in the choroid plexus. Bar=20 μm. e: Representative images of mitochondrial ROS stained with MitoSOX Red in the choroid plexus. Bar=50 μm. f: Statistical results of the damaged mitochondria percentage of total mitochondria according to TEM images (n=3, one-way ANOVA). g: JC-1 staining statistical results reflected MMP (n=3, one-way ANOVA). h: The mitochondrial ROS contents according to MitoSOX Red staining statistical results (n=3, one-way ANOVA). The results are expressed as the mean ± SD, **P < 0.01, *P < 0.05.

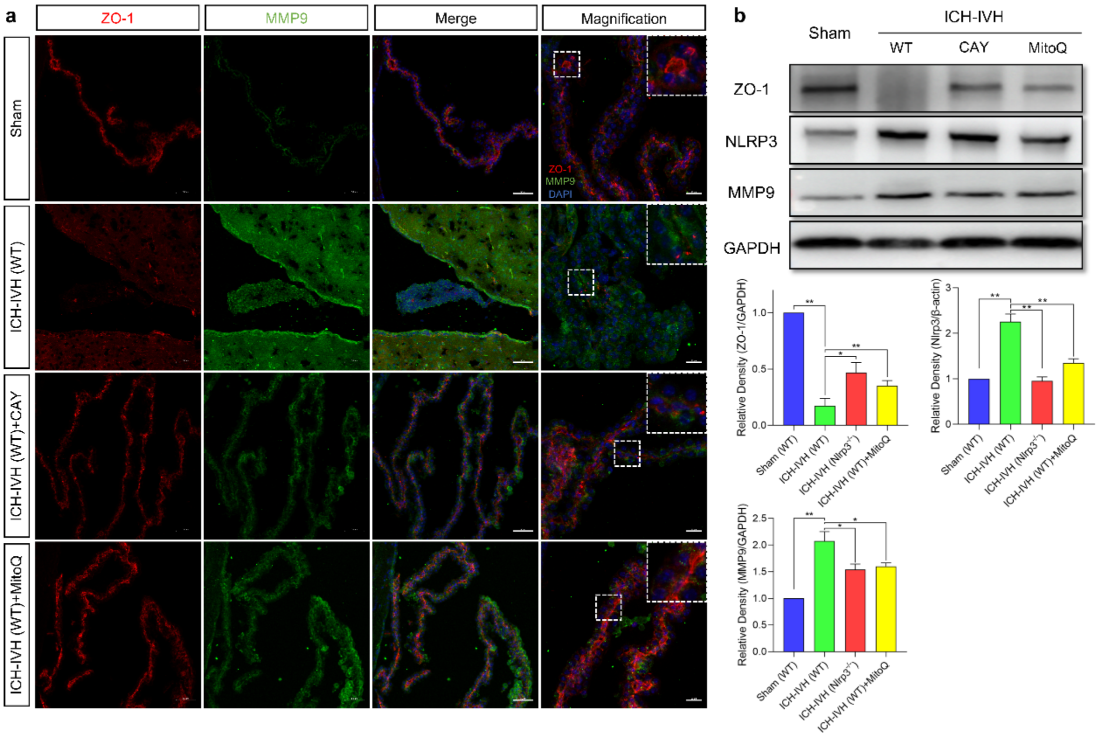

Supplementary Fig. 2. Mitochondrial ROS destroyed the B-CSFB by upregulating MMP9 after ICH-IVH.

a: Immunofluorescence colocalization analysis of ZO-1 and MMP9 in the choroid

plexus after ICH-IVH and CAY10650 or MitoQ treatment. Bar=50 μm. b: The protein expression of ZO-1, NLRP3 and MMP9 in the choroid plexus after ICH-IVH and PLIN3 inhibition or mitochondrial ROS removal (n=3, 6 rats per sample, one-way ANOVA). The results are expressed as the mean ± SD, **P < 0.01, *P < 0.05.

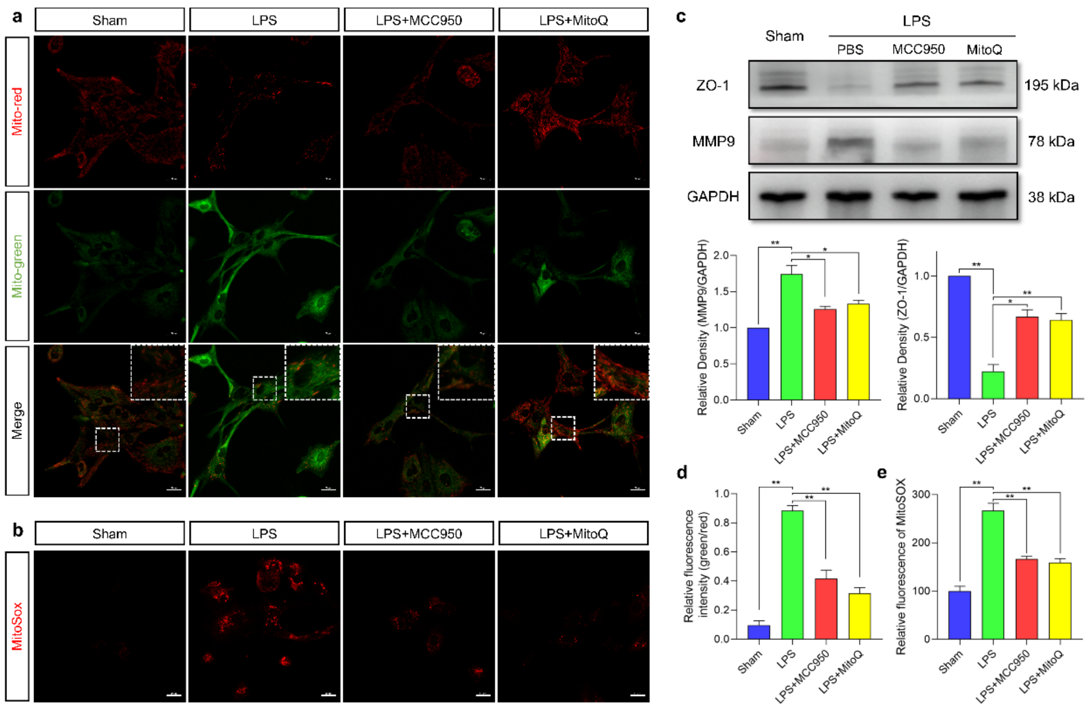

Supplementary Fig. 3. Mitochondrial function and mitochondrial ROS levels after LPS-induced NLRP3 activation in CPECs.

a: JC-1 staining images after LPS-induced NLRP3 activation and CAY10650 or MitoQ treatment. Bar=20 μm. b: MitoSOX Red staining images after different treatments. Bar=20 μm. c: Western blot analysis of ZO-1 and MMP9 in CPECs after LPS stimulation, inhibiting PLIN3 and removing mitochondrial ROS (n=3, one-way ANOVA). d: Statistical results of JC-1 staining reflected MMP changes (n=3, one-way

ANOVA). e: Statistical results of MitoSOX Red staining assessing the content of mitochondrial ROS (n=3, one-way ANOVA). The results are expressed as the mean ± SD, **$P < 0.01$, *$P < 0.05$.

Supplementary Data

Supplementary Table 1-Network images data (Transcriptome)-Figure 4d

Supplementary Table 2-Network images data (Proteome)-Figure 4e
